# Necrostatin-1 Synergizes the Pan Caspase Inhibitor to Attenuate Lung Injury Induced by Ischemia Reperfusion in Rats

**DOI:** 10.1155/2020/7059304

**Published:** 2020-10-24

**Authors:** Liangrong Wang, Baihui Chen, Xiangqing Xiong, Shunli Chen, Lida Jin, Meizhen Zhu

**Affiliations:** Department of Anesthesiology, The First Affiliated Hospital of Wenzhou Medical University, Wenzhou 325000, China

## Abstract

**Background:**

Both apoptosis and necroptosis have been recognized to be involved in ischemia reperfusion-induced lung injury. We aimed to compare the efficacies of therapies targeting necroptosis and apoptosis and to determine if there is a synergistic effect between the two therapies in reducing lung ischemia reperfusion injury.

**Methods:**

Forty Sprague-Dawley rats were randomized into 5 groups: sham (SM) group, ischemia reperfusion (IR) group, necrostatin-1+ischemia reperfusion (NI) group, carbobenzoxy-Val-Ala-Asp-fluoromethylketone+ischemia reperfusion (ZI) group, and necrostatin-1+carbobenzoxy-Val-Ala-Asp-fluoromethylketone+ischemia reperfusion (NZ) group. The left lung hilum was exposed without being clamped in rats from the SM group, whereas the rats were subjected to lung ischemia reperfusion by clamping the left lung hilum for 1 hour, followed by reperfusion for 3 hours in the IR group. 1 mg/kg necrostatin-1 (Nec-1: a specific necroptosis inhibitor) and 3 mg/kg carbobenzoxy-Val-Ala-Asp-fluoromethylketone (z-VAD-fmk: a pan caspase inhibitor) were intraperitoneally administrated prior to ischemia in NI and ZI groups, respectively, and the rats received combined administration of Nec-1 and z-VAD-fmk in the NZ group. Upon reperfusion, expressions of receptor-interacting protein 1 (RIP1), receptor-interacting protein 3 (RIP3), and caspase-8 were measured, and the flow cytometry analysis was used to assess the cell death patterns in the lung tissue. Moreover, inflammatory marker levels in the bronchoalveolar lavage fluid and pulmonary edema were evaluated.

**Results:**

Both Nec-1 and z-VAD-fmk, either alone or in combination, significantly reduced morphological damage, inflammatory markers, and edema in lung tissues following reperfusion, and cotreatment of z-VAD-fmk with Nec-1 produced the optimal effect. The rats treated with Nec-1 had lower levels of inflammatory markers in the bronchoalveolar lavage fluid than those receiving z-VAD-fmk alone (*P* < 0.05). Interestingly, the z-VAD-fmk administration upregulated RIP1 and RIP3 expressions in the lung tissue from the ZI group compared to those in the IR group (*P* < 0.05). Reperfusion significantly increased the percentages of necrotic and apoptotic cells in lung tissue single-cell suspension, which could be decreased by Nec-1 and z-VAD-fmk, respectively (*P* < 0.05).

**Conclusions:**

Nec-1 synergizes the pan caspase inhibitor to attenuate lung ischemia reperfusion injury in rats. Our data support the potential use of Nec-1 in lung transplantation-related disorders.

## 1. Introduction

Lung transplantation is the most curative treatment for many end-stage lung diseases, but early graft failure caused by ischemia reperfusion (IR) remains a formidable obstacle for both early and late survival of lung allografts [1, 2]. Pulmonary dysfunction caused by IR injury is also one of the major complications of perioperative events such as shock, trauma, pulmonary revascularization, and cardiopulmonary bypass. Even though various drugs and techniques have been investigated to reduce IR injury, no effective therapy is currently available to completely eliminate it since the underlying mechanisms are still not fully understood.

Excessive production of reactive oxygen species (ROS) following IR could damage the mitochondrial membrane and stimulate the release of cytochrome c, leading to the initiation of the intrinsic apoptosis pathway [3, 4]. Inhibition of apoptosis could reduce lung injury following IR [5, 6]. Furthermore, necrosis is suggested to be involved in IR-induced lung injury as well [7], but it is not considered as an effective therapeutic target previously, since necrosis is traditionally defined as a nonregulated and accidental type of cell death. However, recent studies have defined a special type of programmed necrosis, termed necroptosis, that can be regulated by multiple pathways [8]. Necroptosis powerfully facilitates inflammation by mediating the inflammatory cytokine secretion and inducing the release of damage-associated molecular patterns (DAMPs), which are involved in many human diseases [8, 9]. Necrostatin-1 (Nec-1), a specific necroptosis inhibitor, decreases pulmonary inflammation and alleviates oleic acid-induced acute respiratory distress syndrome lung injury [10] and also improves survival in neonatal mice with sepsis [11]. Notably, Nec-1 also has been found to attenuate severity of cellular death and mitigate lung injury in experimental lung transplantation [12]. However, the efficacies of therapies targeting necroptosis and apoptosis therapies are not compared, and whether if there is a synergistic effect between the two therapies in reducing lung IR injury has not been determined.

Here, an in situ warm rat lung IR model was established, and the morphological changes in the lung tissue were evaluated. We employed the flow cytometry analysis to confirm the cell death patterns and detected expressions of receptor-interacting proteins (RIPs) and caspase-8 in the lung tissue to evidence the involvements of necroptosis and apoptosis. Moreover, the inflammatory cytokine levels in the bronchoalveolar lavage (BAL) fluid and wet-dry weight (W/D) ratio were measured to indicate pulmonary inflammation and edema, respectively.

## 2. Materials and Methods

Our experimental protocols were approved by the Institutional Animal Care and Use Committee of Wenzhou Medical University (ethic code: FHY2014002) and followed the National Institute of Health Guide for the Care and Use of Laboratory Animals. Forty adult male Sprague-Dawley (SD) rats weighing from 300 to 350 g were purchased from the Experimental Animal Centre of Wenzhou Medical University and maintained for 2 weeks in a licensed, climate-controlled animal room with 12-hour light/12-hour dark cycle. The rats had free access to standard diet and water.

### 2.1. Group Allocation

The rats were randomized into 5 groups (*n* = 8) as follows:

1. Sham group (SM group): the rats received thoracotomy, and the left lung hilum was isolated without being clamped
2. Ischemia reperfusion group (IR group): the rats received thoracotomy, and the left lung hilum subjected to 1-hour ischemia, which was followed by 3-hour reperfusion
3. Nec-1+ischemia reperfusion group (NI group): intraperitoneal administration of 1 mg/kg Nec-1 was given 1 hour before lung ischemia [13], and the remaining procedures were identical to the IR group
4. carbobenzoxy-Val-Ala-Asp-fluoromethylketone+ischemia reperfusion group (ZI group): 3 mg/kg carbobenzoxy-Val-Ala-Asp-fluoromethylketone (z-VAD-fmk) was administrated intraperitoneally 1 hour before ischemia, and the remaining procedures were identical to the IR group
5. Nec-1 + z-VAD-fmk+ischemia reperfusion group (NZ group): the combination of 1 mg/kg Nec-1 and 3 mg/kg z-VAD-fmk was given intraperitoneally 1 hour before the lung ischemia initiation

At the end of experimental protocols, the rats were euthanized by the anesthetic overdose. A midline thoracotomy was performed immediately, and the right main bronchus was ligatured to collect the bronchoalveolar lavage (BAL) fluid from the left lung. After that, the left upper lobe of the lung tissue was obtained for the morphological examination and wet/dry weight ratio (W/D) calculation, the left middle lobe of the lung tissue was used to determine protein expressions of receptor-interacting protein 1 (RIP1), receptor-interacting protein 3 (RIP3) and caspase-8, and the left lower lobe of the lung tissue was collected for single-cell suspension preparation and flow cytometry analysis.

### 2.2. Lung IR Procedure

The rat model of in situ warm lung IR was established using the methods described previously [14]. Briefly, the rats were anesthetized with intraperitoneal administration of ketamine (50 mg/kg) and diazepam (5 mg/kg) [15], and then a 14-gauge angiocatheter was inserted into the trachea through a midline neck incision. The rats were connected to a specialized rodent ventilator (HX-300, Taimeng Inc., Chengdu, China) and ventilated with a tidal volume of 8 ml/kg, respiratory rate of 60/min, I/E ratio of 1 : 2, and inspired oxygen fraction of 0.6. A left thoracotomy was performed at the fifth intercostal space, and the left lung hilum was exposed. After that, 50 units of heparin were administered through the penis vein and allowed to circulate for 5 minutes. A noncrushing clamp was then placed to occlude the left lung hilum for 60 minutes, and the tidal volume was decreased to 2/3 of its original level during ischemia. After 1 hour of ischemia, the clamp was removed, and the left lung was allowed to reventilate with the original tidal volume for up to 3 hours. The chest was temporarily closed during the ischemia and reperfusion period. The rectal temperature was maintained at 37°C with the aid of a warming lamp, and 0.5 ml of warm normal saline was subcutaneously injected hourly to maintain body volume.

### 2.3. BAL Fluid Collection

The left lung was lavaged using 30 ml/kg sterile phosphate buffer saline (PBS) for 3 times, and BAL fluid was then harvested and centrifuged at 3500 g for 10 minutes. BAL fluids were immediately frozen and then stored at -70°C for further detections.

### 2.4. Histological Assessment and Acute Lung Injury Scoring

The lung samples were fixed in 10% formalin and embedded in paraffin, sectioned (4-*μ*m thick), and stained with hematoxylin and eosin. The stained sections were examined and scored by a pathologist blinded to the group assignment using the established acute lung injury scoring system [16].

### 2.5. Electron Microscopic Examination

The lung pieces (1 × 1 mm) were fixed using 2.5% glutaraldehyde in PBS (pH 7.4, 0.1 M) for 24 hours at 4°C. Then, the lung pieces were postfixed in buffered 2% sodium tetroxide for 2 hours, dehydrated in a graded ethanol series, and embedded in epon. The ultrathin sections were stained with uranyl acetate and lead citrate and examined using a transmission electron microscope (H600, Hitachi, Japan).

### 2.6. Water Content in the Lung Tissue

The lung W/D ratio was used as an indicator of pulmonary edema. Immediately after euthanasia, part of the left upper lobe of the lung tissue was weighed to obtain wet weight and then dried at 60°C for 48 hours to determine dry weight. The W/D ratio was defined as wet weight/dry weight.

### 2.7. Flow Cytometry Analysis in Single-Cell Suspension of the Lung Tissue

Single-cell suspensions of the lung tissue were prepared, and the flow cytometry using annexin V- fluorescein isothiocyante (FITC)/propidium iodide (PI) kit was used to detect cell death patterns in the lung tissue. In detail, the dots in the lower left (annexin V-/PI-) quadrant indicated viable cells, those in the upper left (annexin V+/PI+) quadrant were necrotic cells, and those in the lower right (annexin V+/PI-) quadrant were cells undergoing early apoptosis, while those in the upper right (annexin V-/PI+) quadrant were mechanically injured cells.

### 2.8. Protein Expressions of RIP1, RIP3, and Caspase-8

Total protein was extracted from lung homogenates, and Western blot analysis was performed to assess expressions of RIP1, RIP3, and caspase-8. In brief, the target protein (40 mg) was electrophoresed on 10% sodium dodecylsulfate-polyacrylamide gel and transferred to a polyvinylidene difluoride membrane. The membrane was blocked in Tris buffered saline with Tween (TBST; containing 5% nonfat milk, 10 mM Tris, 150 mM NaCl, 0.05% Tween-20) for 1 hour at room temperature and then incubated with first antibodies for RIP1 (1 : 200), RIP3 (1 : 200), caspase-8 (1 : 200), and glyceraldehyde-3-phosphate dehydrogenase (GAPDH; 1 : 5000) at 4°C overnight, respectively. After that, the membrane was incubated with a secondary antibody anti-rabbit IgG (1 : 2000) in TBST solution for 1 hour at room temperature. Blots were developed using the enhanced chemiluminescence system and then scanned. Densitometry was performed with ImageLab Software, and the protein bands were quantified as a ratio to GAPDH control.

### 2.9. Inflammatory Markers and Protein Concentration in the BAL Fluid

The levels of inflammatory marker tumor necrosis factor-*α* (TNF-*α*), interleukin-1*β* (IL-1*β*), interleukin-6 (IL-6), and high-mobility group box 1 (HMGB1) in the BAL fluid were determined with the enzyme-linked immunosorbent assay (ELISA) method according to manufactors' instruments. The protein concentration was determined using a bicinchoninic acid (BCA) protein assay kit.

### 2.10. Statistical Analyses

The data were analyzed with SPSS version 16.0 software. Continuous and normally distributed data were expressed as mean ± standard deviation, and one-way analysis of variance (ANOVA) followed by the post hoc Bonferroni method was used for compare data among groups. A *P* value <0.05 was considered statistically significant.

## 3. Results

### 3.1. Morphological Changes in the Lung Tissue

**Figure 1:**
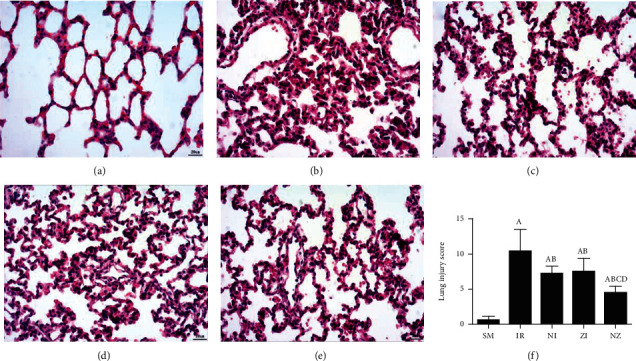
Microscopical changes in the lung tissue. The rats were randomly divided into five groups. Briefly, the rats in the SM group received sham operation, while the left lung hilum was subjected to 1 h of ischemia followed by reperfusion up to 3 h in the IR group. Intraperitoneal Nec-1 (1 mg/kg) and z-VAD-fmk (3 mg/kg) were administrated before lung ischemia in NI and ZI groups, respectively, and combined administration of Nec-1 and z-VAD-fmk was given in the NZ group. After 3 h of reperfusion, the hematoxylin and eosin-stained lung sections were examined under light microscope, and the lung injury score was also evaluated. Figures 1(a)–1(e) are representative photomicrographs (at ×400 magnifications) of SM, IR, NI, ZI, and NZ groups, respectively, and Figure 1(f) shows the lung injury score in groups. Data were presented as mean ± SD, *n* = 8 in each group. ^a^*P* < 0.05 compared with the SM group, ^b^*P* < 0.05 compared with the IR group, ^c^*P* < 0.05 compared with the NI group, ^d^*P* < 0.05 compared with the ZI group.

**Figure 2:**
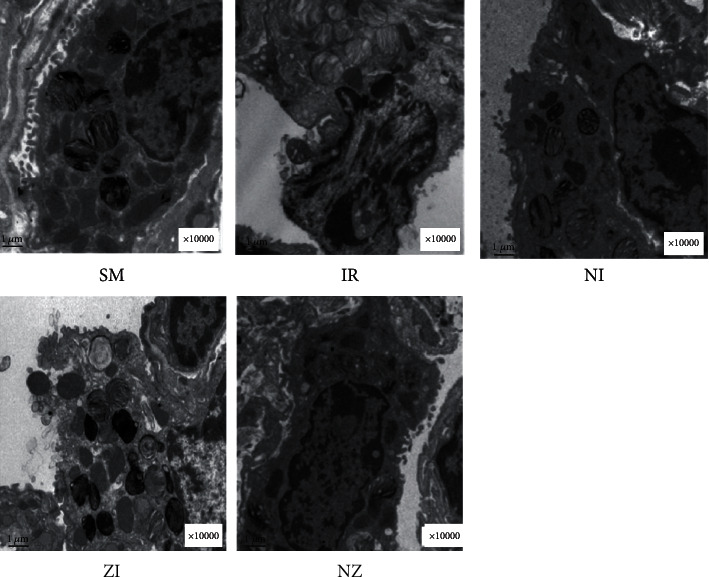
Ultrastructural changes in the lung tissue. The rats were randomly divided into five groups. Briefly, the rats in the SM group received sham operation, while the left lung hilum was subjected to 1 h of ischemia followed by reperfusion up to 3 h in the IR group. Intraperitoneal Nec-1 (1 mg/kg) and z-VAD-fmk (3 mg/kg) were administrated before lung ischemia in NI and ZI groups, respectively, and combined administration of Nec-1 and z-VAD-fmk was given in the NZ group. After 3 h of reperfusion, lung sections were examined under electron microscope.  Figure 2 is a representative photomicrograph of different groups, and images are at ×10000 magnifications.

Normal microstructures and ultrastructures of the lung tissue were observed in the SM group. Lung tissues in the IR group were severely damaged, demonstrated by obvious alveolar wall thickening, interstitial edema, and neutrophil infiltration by hematoxylin and eosin staining. The electron microscopic examination showed swollen mitochondria, disordered cristae, vacuolized cytoplasmic lamellar bodies, partly falling microvilli, and disrupted cell membrane integrity in type II alveolar epithelial cells from the IR group. Morphological damages in the lung tissue were significantly alleviated in both NI and ZI groups, but the rats in the NZ group showed the most improved morphological structures compared with the IR group (Figures 1(a)–1(e) and 2).

The lung injury scores paralleled the findings of the histological examination, and the lung injury scores of the IR group were significantly higher than those of the SM group, whereas the lung injury scores of either NI or ZI group were significantly lower than those of the IR group (*P* < 0.05). As expected, the rats in the NZ group had the lowest acute lung injury scores among these groups (*P* < 0.05, Figure 1(f)).

### 3.2. Water Content in the Lung Tissue

**Figure 3:**
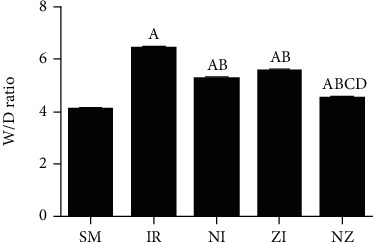
Water content in the lung tissue. The rats were randomly divided into five groups. Briefly, the rats in the SM group received sham operation, while the left lung hilum was subjected to 1 h of ischemia followed by reperfusion up to 3 h in the IR group. Intraperitoneal Nec-1 (1 mg/kg) and z-VAD-fmk (3 mg/kg) were administrated before lung ischemia in NI and ZI groups, respectively, and combined administration of Nec-1 and z-VAD-fmk was given in the NZ group. After 3 h of reperfusion, lung edema was assessed by the W/D ratio, W/D ratio, and wet/dry weight ratio. Data were presented as mean ± SD, *n* = 8 in each group. ^a^*P* < 0.05 compared with the SM group, ^b^*P* < 0.05 compared with the IR group, ^c^*P* < 0.05 compared with NI and ZI group.

As shown in Figure 3, W/D ratios in the lung tissue were significantly higher in the IR group than the SM group (*P* < 0.05), which suggested pulmonary edema after IR. Consistent with the histological findings, lung W/D ratios in both NI and ZI groups were lower than the IR group, but higher than the NZ group (*P* < 0.05).

### 3.3. Cell Death in Single-Cell Suspension of the Lung Tissue

**Figure 4:**
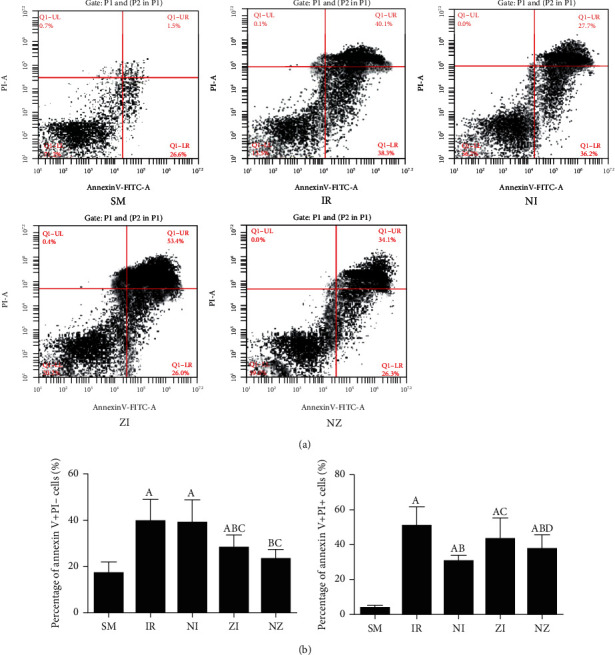
Flow cytometry analysis in lung tissue single-cell suspension. The rats were randomly divided into five groups. Briefly, the rats in the SM group received sham operation, while the left lung hilum was subjected to 1 h of ischemia followed by reperfusion up to 3 h in the IR group. Intraperitoneal Nec-1 (1 mg/kg) and z-VAD-fmk (3 mg/kg) were administrated before lung ischemia in NI and ZI groups, respectively, and combined administration of Nec-1 and z-VAD-fmk was given in the NZ group. After 3 h of reperfusion, single-cell suspension of the lung tissue was prepared, and cell death was assessed using flow cytometry. Figures 4(a) and 4(b) are representative photomicrographs of different groups. Data were presented as mean ± SD, *n* = 8 in each group. ^a^*P* < 0.05 compared with the SM group, ^b^*P* < 0.05 compared with the IR group, ^c^*P* < 0.05 compared with the NI group, ^d^*P* < 0.05 compared with the ZI group.

IR remarkably induced necrotic and apoptotic cell death in single-cell suspension of the lung tissue, which were evidenced by increased percentages of both annexin V+/PI- and annexin V+/PI+ cells (*P* < 0.05). As shown in Figure 4, the percentages of annexin V+/PI+ cells in the NI group and annexin V+/PI- cells in the ZI group were significantly lower than those in the IR group (*P* < 0.05), and combined treatment of Nec-1 and z-VAD-fmk significantly reduced the percentages of both necrotic and apoptotic cells (*P* < 0.05). Moreover, the rats treated with z-VAD-fmk had larger percentage of annexin V+/PI+ cells in single-cell suspension of the lung tissue compared with those treated with Nec-1 (*P* < 0.05).

### 3.4. RIP1, RIP3, and Caspase-8 Expressions in the Lung Tissue

**Figure 5:**
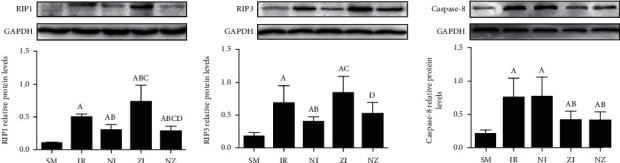
Protein expressions of RIP1, RIP3, and caspase-8. The rats were randomly divided into five groups. Briefly, the rats in the SM group received sham operation, while the left lung hilum was subjected to 1 h of ischemia followed by reperfusion up to 3 h in the IR group. Intraperitoneal Nec-1 (1 mg/kg) and z-VAD-fmk (3 mg/kg) were administrated before lung ischemia in NI and ZI groups, respectively, and combined administration of Nec-1 and z-VAD-fmk was given in the NZ group. After 3 h of reperfusion, protein expressions of RIP1, RIP3, and caspase-8 were measured by Western bolt. RIP1: receptor-interacting protein 1; RIP3: receptor-interacting protein 3. Data were presented as mean ± SD, *n* = 8 in each group. ^a^*P* < 0.05 compared with the SM group, ^b^*P* < 0.05 compared with the IR group, ^c^*P* < 0.05 compared with the NI group, ^d^*P* < 0.05 compared with the ZI group.

As shown in Figure 5, IR upregulated RIP1, RIP3, and caspase-8 expressions in the lung tissue (*P* < 0.05). The expressions of RIP1 and RIP3, but not caspase-8, were reduced by Nec-1 treatment, while the z-VAD-fmk administration downregulated the caspase-8 expression and meanwhile, increased RIP1 and RIP3 expressions in the lung tissue responding to IR injury (*P* < 0.05). The combined administration of Nec-1 and z-VAD-fmk decreased expressions of these three proteins compared with the IR group (*P* < 0.05).

### 3.5. Inflammatory Markers, HMGB1, and Protein Concentrations in the BAL Fluid

**Figure 6:**
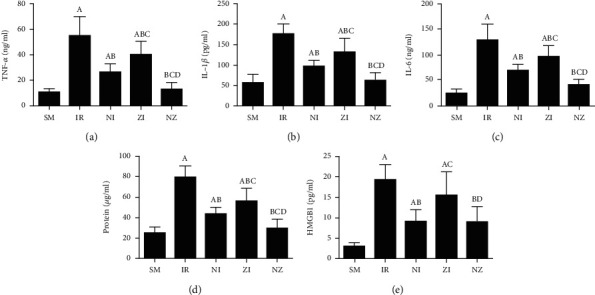
Inflammatory markers and protein concentration in the BAL fluid. The rats were randomly divided into five groups. Briefly, the rats in the SM group received sham operation, while the left lung hilum was subjected to 1 h of ischemia followed by reperfusion up to 3 h in the IR group. Intraperitoneal Nec-1 (1 mg/kg) and z-VAD-fmk (3 mg/kg) were administrated before lung ischemia in NI and ZI groups, respectively, and combined administration of Nec-1 and z-VAD-fmk was given in the NZ group. After 3 h of reperfusion, the BAL fluid was harvested, and the levels of TNF-*α*, IL-1*β*, IL-6, protein, and HMGB1 in the BAL fluid were detected. BAL fluid: bronchoalveolar lavage fluid; TNF-*α*: tumor necrosis factor-*α*; IL-1*β*: interleukin-1*β*; IL-6: interleukin-6; HMGB1: high-mobility group box 1. Data were presented as mean ± SD, *n* = 8 in each group. ^a^*P* < 0.05 compared with the SM group, ^b^*P* < 0.05 compared with the IR group, ^c^*P* < 0.05 compared with the NI group, ^d^*P* < 0.05 compared with the ZI group.

Figure 6 demonstrates the levels of inflammatory markers and protein in the BAL fluid. As compared with the SM group, the rats that underwent lung IR injury had much higher levels of inflammatory markers and protein in the BAL fluid (*P* < 0.05), which could be partly suppressed by the treatment of Nec-1 or z-VAD-fmk alone (*P* < 0.05), and the rats receiving combined treatment of the two agents had the lowest inflammatory markers and protein levels in the BAL fluid among these groups (*P* < 0.05). Furthermore, the elevated level of HMGB1 in the BAL fluid in the IR group was decreased by the treatment of Nec-1 but not z-VAD-fmk (*P* < 0.05). Importantly, the levels of inflammatory markers and protein were reduced in the NI group compared with the ZI group (*P* < 0.05), and HMGB1 levels in the BAL fluid did not differ between NI and NZ groups (*P* > 0.05).

## 4. Discussion

The main findings of our present study were that (1) necroptosis, a newly recognized cell death type, was involved in IR-induced lung injury; (2) treatment with the pan caspase inhibitor z-VAD-fmk alleviated lung IR injury, but meanwhile potentially facilitated RIP-mediated necroptosis; and (3) the specific necroptosis inhibitor Nec-1 was more effective in attenuating the inflammatory response, which would synergize z-VAD-fmk to reduce IR-induced lung injury in rats.

Reoxygenation to previously ischemic tissue results in formation of highly toxic ROS through several key mechanisms that generally included activation of nicotinamide adenine dinucleotide phosphate oxidase and xanthine oxidase and mitochondrial dysfunction [17]. Excessively produced ROS would damage various cellular components, where nucleic acids in DNA or RNA, amino acids in proteins, and fatty lipids in the cell membrane are especially at risk. Subsequently, ROS causes multiple patterns of cell death and tissue damage [4, 18]. Flow cytometry using annexin V and PI staining provides a rapid and convenient assay for apoptosis and necrosis, the two classical modes of cell death [19]. PI is not a cell-permeable fluorescent dye, and it specifically binds to nucleic acids to stain the nuclei of the dead cells with disrupted plasma membrane. While, annexin V is a calcium-dependent phospholipid-binding protein with a high affinity to phosphatidylserine, it can bind to externalized phosphatidylserine of the apoptotic cell. Therefore, fluorescein-labeled annexin V-positive (annexin V+PI-) cells could be recognized as apoptotic ones, while annexin V and PI double-positive staining (annexin V+PI+) indicate necrosis. Here, we found that IR significantly induced both apoptotic and necrotic cell death in the lung tissue. Caspase-dependent apoptosis is considered as a classical contributor to IR-induced lung injury, and the effectiveness of antiapoptotic therapies has been well demonstrated in previous studies [5, 6, 20, 21]. In this study, z-VAD-fmk, a pan caspase inhibitor, significantly reduced apoptotic cell death and pathological severity in the lung tissue and decreased inflammatory markers and protein levels in BAL fluid as well. Taken together, these results again evidenced the involvement of apoptosis in IR-induced lung injury.

Necroptosis is a special programmed cell death pattern that was proposed in recent years, and it is recognized to be mediated by kinases RIP1 and RIP3 [8, 22, 23]. Necroptosis shares similar morphologic features with necrosis, and accumulating evidences have highlighted its importance in mediating various pathophysiologic events [12, 24–29]. Mixed lineage kinase domain-like protein (MLKL) is identified as a key component downstream of RIP3 in the execution of RIP1-mediated necroptosis, and MLKL trimerization and translocation facilitate the channel formation on the plasma membrane through which DAMPs are released [21, 30, 31]. Thus, necroptosis is typically characterized by cellular swelling and rupture, and the loss of cellular membrane integrity would promote inflammation via the release of DAMPs into the extracellular environment [22, 32]. Nec-1, a specific small molecule capable of inhibiting RIP1 kinase activity, blocks the progress of the downstream necrotic events [33]. The efficacy of this small molecule has been demonstrated in reducing ROS generation, modulating the inflammatory pathway, and decreasing necrotic cell death in several models of IR and organ transplantation [24, 27, 28, 34, 35].

Here, IR caused lung tissue damages along with necrotic cell death and increased expressions of RIPs, which could be significantly attenuated by Nec-1 treatment. Moreover, the Nec-1 administration also reduced protein leakage that indicated impaired cellular membrane and the formation of inflammatory mediators. HMGB1, a well-recognized necroptosis marker [36] was significantly increased in the BAL fluid following IR and could be reduced by Nec-1 but not z-VAD-fmk treatment. These data supported the contribution of necroptosis to lung IR injury, and this was consistent with the results by Kanou T et al., who demonstrated that the Nec-1 administration to both donor and recipient could significantly inhibit necrotic cell death and improve graft function following lung transplantation in rats [12]. In the present study, Nec-1 and z-VAD-fmk shared similar effects in reducing pulmonary morphological abnormalities and edema induced by IR, but the Nec-1 administration was more effective than v-ZAD-fmk in protecting against inflammation and disrupted cellular membranes in our experimental lung IR.

Our present study showed that z-VAD-fmk did reduce apoptotic cell death and downregulated the caspase-8 expression, but z-VAD-fmk facilitated expressions of RIP1 and RIP3 and paradoxically increased necrotic cell death in lung tissues responding to IR compared with those treated with Nec-1. Mechanically, activation of caspase-8 inhibits necroptosis by cleavage of RIP1, but when caspase-8 is genetically depleted or inhibited, RIP1 interacts with RIP3 and MLKL upon death receptor activation to form a necrosome complex [8, 31, 32]. Thus, using inhibitors of caspases or genetic ablation of caspase-8 has been demonstrated to switch cell fate from apoptosis to necroptosis [31, 37]. Our data demonstrated that Nec-1 remarkably diminished the unfavorable effect of pan caspase inhibitor on facilitating necrotic cell death and shared the synergy with z-VAD-fmk to reduce lung IR injury.

Our study had certain clinical implication that inhibition of necroptosis, added to a pan caspase inhibitor, seems to be a more promising strategy for preventing IR lung injury in future clinical settings. Further studies are needed to explore the detailed mechanisms or underlying pathways in animal study or replicate our findings in human subjects when agents are clinically available. There are other limitations that should be addressed. Firstly, owing to the lack of reliable antibodies of phospho-MLKL, we failed to trace the downstream pathway of RIP1 and RIP3, and future studies are necessary when commercial antibodies are produced. Secondly, it would be more convincing if in vitro data are provided to support the findings in this animal study. Finally, the protective effect of a single dose of Nec-1 was tested in our study, and the dose-response data are needed to identify the “ideal” dose.

## 5. Conclusions

**N**ecroptosis contributes to IR-induced lung injury, and inhibition of necroptosis with Nec-1 synergizes the pan caspase inhibitor to attenuate lung IR injury in rats. Our data support the promising use of Nec-1 in lung transplantation-related disorders.

## Data Availability

All data included in this study are available upon request by contacting with the corresponding author.

## References

[1] Chen F., Date H. (2015). Update on ischemia-reperfusion injury in lung transplantation. *Current Opinion in Organ Transplantation*.

[2] Porteous M. K., Lee J. C. (2017). Primary graft dysfunction after lung transplantation. *Clinics in Chest Medicine*.

[3] Soltani M., Moghimian M., Abtahi-Eivari S. H., Shoorei H., Khaki A., Shokoohi M. (2018). Protective effects of Matricaria chamomilla extract on torsion/ detorsion-induced tissue damage and oxidative stress in adult rat testis. *International Journal of Fertility & Sterility*.

[4] Moghimian M., Soltani M., Abtahi H., Shokoohi M. (2017). Effect of vitamin C on tissue damage and oxidative stress following tunica vaginalis flap coverage after testicular torsion. *Journal of Pediatric Surgery*.

[5] Zhu B., Yang J., Chen S. (2017). Oxymatrine on Hsp90a expression and apoptosis in a model of lung ischemia-reperfusion injury. *Experimental and Therapeutic Medicine*.

[6] Liu W. C., Chen S. B., Liu S. (2019). Inhibition of mitochondrial autophagy protects donor lungs for lung transplantation against ischaemia-reperfusion injury in rats via the mTOR pathway. *Journal of Cellular and Molecular Medicine*.

[7] Kim H., Zhao J., Zhang Q. (2015). *δ*V1‐1 reduces pulmonary ischemia reperfusion-induced lung injury by inhibiting necrosis and mitochondrial localization of PKC*δ* and p53. *American Journal of Transplantation*.

[8] Galluzzi L., Kroemer G. (2008). Necroptosis: a specialized pathway of programmed necrosis. *Cell*.

[9] Khoury M. K., Gupta K., Franco S. R., Liu B. (2020). Necroptosis in the pathophysiology of disease. *The American Journal of Pathology*.

[10] Pan L., Yao D. C., Yu Y. Z. (2016). Necrostatin-1 protects against oleic acid-induced acute respiratory distress syndrome in rats. *Biochemical and Biophysical Research Communications*.

[11] Bolognese A. C., Yang W. L., Hansen L. W. (2018). Inhibition of necroptosis attenuates lung injury and improves survival in neonatal sepsis. *Surgery*.

[12] Kanou T., Ohsumi A., Kim H. (2018). Inhibition of regulated necrosis attenuates receptor-interacting protein kinase 1-mediated ischemia-reperfusion injury after lung transplantation. *The Journal of Heart and Lung Transplantation*.

[13] Li X., Ling Y., Cao Z. (2018). Targeting intestinal epithelial cell-programmed necrosis alleviates tissue injury after intestinal ischemia/reperfusion in rats. *The Journal of Surgical Research*.

[14] Eppinger M. J., Ward P. A., Jones M. L., Bolling S. F., Deeb G. M. (1995). Disparate effects of nitric oxide on lung ischemia-reperfusion injury. *The Annals of Thoracic Surgery*.

[15] Yi-Ming W., Shu H., Miao C. Y., Shen F. M., Jiang Y. Y., Su D. F. (2004). Asynchronism of the recovery of baroreflex sensitivity, blood pressure, and consciousness from anesthesia in rats. *Journal of Cardiovascular Pharmacology*.

[16] Yang C. H., Tsai P. S., Wang T. Y., Huang C. J. (2009). Dexmedetomidine-ketamine combination mitigates acute lung injury in haemorrhagic shock rats. *Resuscitation*.

[17] Granger D. N., Kvietys P. R. (2015). Reperfusion injury and reactive oxygen species: the evolution of a concept. *Redox Biology*.

[18] Shokoohi M., Khaki A., Shoorei H., Khaki A. A., Moghimian M., Abtahi-Eivary S. H. (2019). Hesperidin attenuated apoptotic-related genes in testicle of a male rat model of varicocoele. *Andrology*.

[19] Vermes I., Haanen C., Steffens-Nakken H., Reutellingsperger C. (1995). A novel assay for apoptosis. Flow cytometric detection of phosphatidylserine expression on early apoptotic cells using fluorescein labelled annexin V. *Journal of Immunological Methods*.

[20] (2019). Mesenchymal stromal cells-derived exosomes alleviate ischemia/reperfusion injury in mouse lung by transporting anti-apoptotic miR-21-5p. *European Journal of Pharmacology*.

[21] Tian W. F., Weng P., Sheng Q. (2017). Biliverdin protects the isolated rat lungs from ischemia-reperfusion injury via antioxidative, anti-inflammatory and anti-apoptotic effects. *Chinese Medical Journal*.

[22] Pasparakis M., Vandenabeele P. (2015). Necroptosis and its role in inflammation. *Nature*.

[23] Silke J., Rickard J. A., Gerlic M. (2015). The diverse role of RIP kinases in necroptosis and inflammation. *Nature Immunology*.

[24] Linkermann A., Bräsen J. H., Himmerkus N. (2012). Rip1 (receptor-interacting protein kinase 1) mediates necroptosis and contributes to renal ischemia/reperfusion injury. *Kidney International*.

[25] Zhang T., Zhang Y., Cui M. (2016). CaMKII is a RIP3 substrate mediating ischemia- and oxidative stress-induced myocardial necroptosis. *Nature Medicine*.

[26] Linkermann A., Hackl M. J., Kunzendorf U., Walczak H., Krautwald S., Jevnikar A. M. (2013). Necroptosis in immunity and ischemia-reperfusion injury. *American Journal of Transplantation*.

[27] Oerlemans M. I. F. J., Liu J., Arslan F. (2012). Inhibition of RIP1-dependent necrosis prevents adverse cardiac remodeling after myocardial ischemia-reperfusion in vivo. *Basic Research in Cardiology*.

[28] Wen S., Ling Y., Yang W. (2017). Necroptosis is a key mediator of enterocytes loss in intestinal ischaemia/reperfusion injury. *Journal of Cellular and Molecular Medicine*.

[29] Mizumura K., Maruoka S., Gon Y., Choi A. M. K., Hashimoto S. (2016). The role of necroptosis in pulmonary diseases. *Respiratory Investigation*.

[30] Rodriguez D. A., Weinlich R., Brown S. (2016). Characterization of RIPK3-mediated phosphorylation of the activation loop of MLKL during necroptosis. *Cell Death and Differentiation*.

[31] Someda M., Kuroki S., Miyachi H., Tachibana M., Yonehara S. (2020). Caspase-8, receptor-interacting protein kinase 1 (RIPK1), and RIPK3 regulate retinoic acid-induced cell differentiation and necroptosis. *Cell Death and Differentiation*.

[32] Galluzzi L., Kepp O., Chan F. K. M., Kroemer G. (2017). Necroptosis: mechanisms and relevance to disease. *Annual Review of Pathology*.

[33] Degterev A., Huang Z., Boyce M. (2005). Chemical inhibitor of nonapoptotic cell death with therapeutic potential for ischemic brain injury. *Nature Chemical Biology*.

[34] Yang R., Hu K., Chen J. (2017). Necrostatin-1 protects hippocampal neurons against ischemia/reperfusion injury via the RIP3/DAXX signaling pathway in rats. *Neuroscience Letters*.

[35] Wang X., O’Brien M. E., Yu J. (2019). Prolonged cold ischemia induces necroptotic cell death in ischemia-reperfusion injury and contributes to primary graft dysfunction after lung transplantation. *American Journal of Respiratory Cell and Molecular Biology*.

[36] Xu Z., Jin Y., Yan H. (2018). High-mobility group box 1 protein-mediated necroptosis contributes to dasatinib-induced cardiotoxicity. *Toxicology Letters*.

[37] Fritsch M., Günther S. D., Schwarzer R. (2019). Caspase-8 is the molecular switch for apoptosis, necroptosis and pyroptosis. *Nature*.

